# Lemon Balm ( *Melissa officinalis* L.) Leaf Extract Promotes Endo180 Production in Dermal Fibroblasts and has Antiwrinkle Effect on Human Skin

**DOI:** 10.1111/phpp.70006

**Published:** 2025-01-31

**Authors:** Hiroyasu Iwahashi, Yoshihito Kawashima, Hitoshi Masaki, Atsushi Taga

**Affiliations:** ^1^ Research Center Maruzen Pharmaceuticals Co., Ltd. Hiroshima Japan; ^2^ Laboratory of Photoaging Research, School of Bioscience and Biotechnology Tokyo University of Technology Tokyo Japan; ^3^ Research Institute for Human Health Science Konan University Hyougo Japan; ^4^ Pathological and Biomolecule Analyses Laboratory, Faculty of Pharmacy Kindai University Osaka Japan; ^5^ Antiaging Center Kindai University Osaka Japan

**Keywords:** Endo180, *Melissa officinalis*, photoaging, type I collagen, wrinkle

## Abstract

**Background:**

The collagen receptor Endo180 participates in extracellular matrix remodeling by clearing the pericellular environment and recognizing and internalizing collagen degradation products. In photoaged skin, Endo180 expression in fibroblasts is decreased, and collagen fragments accumulate in the pericellular environment, leading to a decrease in type I collagen production and an increase in matrix metalloproteinase 1 production. This suggests that a decrease in Endo180 production may promote wrinkle formation by decreasing the dermal collagen fibril volume. Therefore, this study aimed to identify materials that promote Endo180 production in vitro and investigate whether promoting Endo180 production could prevent and improve wrinkles in vivo.

**Methods:**

Endo180 gene expression and protein production in fibroblasts were evaluated after screening 71 natural extracts. The conditioned medium of UVB‐irradiated keratinocytes and Endo180 production‐promoting extract were added to fibroblasts, and Endo180 and type I collagen production were evaluated. In a double‐blind, randomized, placebo‐controlled study, a cream formulated with an Endo180 production‐promoting extract or placebo was topically administered to each side of the face of 20 healthy women twice daily for 8 weeks.

**Results:**

Screening results showed that 50 μg/mL of lemon balm ( *Melissa officinalis* L.) leaf extract (MOLE) resulted in the highest levels of both Endo180 mRNA and protein at 178.1% and 127.4%, respectively. Its major component rosmarinic acid also promoted Endo180 production by 143.9% at a concentration of 20 μg/mL. MOLE at 200 μg/mL almost completely inhibited the decrease in Endo180 and type I collagen production in UVB‐irradiated keratinocyte‐conditioned medium. Furthermore, eye‐corner wrinkles were reduced by treatment with the MOLE formulation compared to that in response to the placebo formulation.

**Conclusions:**

MOLE may act as an antiwrinkle agent that inhibits the decline in collagen levels by promoting Endo180 production.

## 1 Introduction

The transmembrane protein Endo180 (alias mannose receptor C Type 2: MRC2/urokinase plasminogen activator receptor‐associated protein: uPARAP/CD280), a collagen uptake receptor, is involved in collagen remodeling via extracellular collagen uptake [1]. The collagen fiber structure is maintained by the metabolic balance of collagen fiber synthesis and degradation, known as collagen remodeling. The degradation process can be roughly divided into the fragmentation of collagen fibers by matrix metalloproteinases (MMPs) and the uptake of fragmented collagen through Endo180 by fibroblasts. Endo180 possesses the ability to take up fragments of collagen types I, IV, and V [2]. For type I collagen, denatured collagen such as gelatin is more accessible to Endo180 than intact collagen [3]. Denatured and fragmented collagen is degraded to amino acids by proteolytic enzymes in the lysosome after uptake into fibroblasts [4]. Thus, the recycling process of collagen through Endo180 is important for maintaining extracellular matrix homeostasis.

In photoaged skin caused by prolonged exposure to ultraviolet (UV) radiation, changes in the dermal matrix structure such as the loss of collagen and oxytalan fibers in the papillary dermis and the accumulation of large elastic fibers in the reticular dermis are well established [5]. The loss of type I collagen fibers, acting as dermal skeletal fibers, plays a crucial role in the progression of photoaging [6]. The expression of Endo180 in fibroblasts has been reported to be decreased in photoaged dermis. Consequently, collagen fragments accumulate around the cell, affecting wrinkle formation [7]. It has been demonstrated that Endo180 production was reduced directly by UVA exposure to fibroblasts or indirectly via interleukin 1α produced by UVB‐exposed keratinocytes [8, 9]. Fibroblasts with reduced Endo180 without the influence of UV exposure do not take up denatured collagen, and it instead accumulates in the surrounding area, leading to increased reactive oxygen species (ROS) production [10]. ROS suppress type I collagen production and promote MMP‐1 production, resulting in accumulation of its degraded products around the fibroblast by enhancement of new collagen degradation. The decreased level of Endo180 is the starting point of a negative loop.

Thus, it is considered that reducing Endo180 affects wrinkle formation through the alterations in the dermal matrix [10]. Therefore, promoting the expression of Endo180 or suppressing the decrease in the expression of Endo180 may be one way to suppress or improve wrinkling by preventing collagen loss. However, there is no direct evidence, and there are few reports on substances that promote Endo180 expression.

This study aimed to search for natural extracts that increase the production of Endo180 in dermal fibroblasts and to clarify whether the lemon balm ( *Melissa officinalis* L.) leaf extract (MOLE) chosen by screening for its promoting effect on Endo180 production improves wrinkles by recovering reduced collagen production and normalizing collagen remodeling through the increased production of Endo180.

## 2 Materials and Methods

### 2.1 Chemicals and Reagents

NB1RGB cells (RIKEN BioResource Center, Ibaraki, Japan), Dulbecco's modified Eagle medium (DMEM), Hanks' balanced salt solution (HBSS; Nissui Pharmaceutical, Tokyo, Japan), fetal bovine serum (FBS; Biosera, Nuaille, France), penicillin/streptomycin, rosmarinic acid, and 10% formalin neutral buffer solution (Wako Pure Chemical Corporation, Osaka, Japan), trypsin/EDTA (Thermo Fisher Scientific, Waltham, MA, USA), ISOGEN (Nippon Gene, Tokyo, Japan), PrimeScript RT Master Mix, TB Green Fast qPCR Mix, the primer set for Endo180 (*MRC2*), and glyceraldehyde‐3‐phosphate dehydrogenase (*GAPDH*; Takara Bio, Shiga, Japan), Block Ace (DS Pharma Biomedical, Osaka, Japan), the mouse anti‐human Endo180 antibody (Abnova, Taipei, Taiwan), horseradish peroxidase (HRP)‐conjugated anti‐mouse IgG antibody (Santa Cruz Biotechnology, Texas, USA), rabbit anti‐human type I collagen antibody and recombinant type I collagen (ROCKLAND, Gilbertsville, PA, USA), HRP‐conjugated anti‐rabbit IgG antibody (Jackson ImmunoResearch, West Grove, PA, USA), bovine serum albumin (BSA), 2,2′‐azino‐bis(3‐ethylbenzothiazoline‐6‐sulfonate) (ABTS; Merck, Darmstadt, Germany), and Hoechst33342 (Dojindo, Kumamoto, Japan) were obtained from various suppliers. All other chemicals and reagents used in this study were of analytical grade.

### 2.2 Extract Preparation

Seventy‐one freeze‐dried natural extracts (Table S1) prepared by Maruzen Pharmaceuticals (Hiroshima, Japan) were screened. To prepare MOLE, lemon balm (*M. officinalis*) leaves (200 g) were extracted with 3.2 L of 50 vol% 1,3‐butylene glycol solution at 80°C–90°C for 3 days. The mixture was then filtered through diatomite. The filtrate was concentrated under reduced pressure at 60°C. The residue was then freeze dried, providing 52 g of MOLE (yield 26%) that was stored in the dark at −30°C until use. Each sample was dissolved in DMSO to prepare a 50 mg/mL solution and diluted to the indicated concentrations, resulting in less than 0.5% DMSO. The same dose of DMSO without the sample was used as the control.

### 2.3 Cell Culture

NB1RGB cells and HaCaT keratinocytes were cultured in DMEM containing 10% FBS, 100 U/mL penicillin, and 100 μg/mL streptomycin. Cell cultures were passaged using trypsin/ethylenediaminetetraacetic acid and maintained at 37°C with 5% CO_2_.

### 2.4 Quantitative Reverse Transcription PCR (qRT‐PCR) for Screening

Gene expression in NB1RGB (1.0 × 10^6^ cells/60 mm dish) cells seeded in a medium with 5 or 50 μg/mL screening sample for 24 h was analyzed using qRT‐PCR. Total RNA was isolated from cells using ISOGEN according to the standard operating method, and cDNA was synthesized using the PrimeScript RT Master Mix. Target mRNA expression was quantitatively analyzed using cDNA templates in a real‐time PCR assay performed with the TB Green Fast qPCR Mix. The mRNA levels were quantified using the calibration curve method. Endo180 mRNA expression was normalized to *GAPDH* and expressed as a percentage of the control cells.

### 2.5 Endo180 Enzyme‐Linked Immunosorbent Assay (ELISA) for Screening

The protein level of Endo180 on the cell membrane in NB1RGB (1.0 × 10^5^ cells/well in 96‐well plates) cells seeded into a medium with 5 or 50 μg/mL screening sample for 24 h was evaluated by ELISA. After culturing, cells were fixed in 10% formalin neutral buffer for 30 min at 25°C. After blocking with Block Ace, the cells were incubated with mouse anti‐human Endo180 antibody for 2 h at room temperature and then incubated with HRP‐conjugated anti‐mouse IgG antibody for 1 h at 25°C. ABTS was used as a substrate for HRP, and color development was evaluated. The absorbance at 405 nm was measured using a microplate reader. The amount of Endo180 protein was expressed as a percentage of that in control cells.

### 2.6 Endo180 ELISA for MOLE and Rosmarinic Acid

After incubating NB1RGB cells seeded at 1.0 × 10^5^ cells/well in 96‐well plates in DMEM containing 0.25% FBS with the indicated concentrations of MOLE or rosmarinic acid for 24 h, the protein levels of Endo180 on the cell membrane and in the whole cell were measured using the same ELISA method as described above. When measuring Endo180 levels in whole cells, an additional 15 min of membrane permeabilization with 0.5% saponin in phosphate‐buffered saline (PBS) was performed. A calibration curve was plotted using the Endo180 recombinant protein, and the concentration of Endo180 was calculated. The number of cells was determined by measuring the fluorescence of nuclei stained with Hoechst 33342 (excitation 352 nm and emission 461 nm). Endo180 protein levels were normalized by dividing the Endo180 concentration by the Hoechst 33342 fluorescence. The results were expressed as a percentage of the level in control cells.

### 2.7 HPLC Analysis of Rosmarinic Acid

Quantitative analysis of rosmarinic acid was performed using a liquid chromatography system (Nexera lite, SHIMADZU CORPORATION, Kyoto, Japan) equipped with a UV–VIS detector SPD‐M40 and a reverse‐phase column YMC‐Pack Pro C18 (4.6 × 150 mm, 5 μm; YMC, Kyoto, Japan). The oven temperature was 40°C. The mobile phase was water/acetonitrile/trifluoroacetic acid (1000:250:1 *v*/*v*/*v*). The flow rate was 1.0 mL/min, and the injection volume was 20 μL. UV spectra were collected over the range 190–400 nm. Chromatograms at 330 nm were obtained using the LabSolutions software. The rosmarinic acid standard was dissolved with the mobile phase to obtain 2.09, 20.9, and 209 μg/mL solutions. MOLE (50 mg/mL) in DMSO was dissolved in the mobile phase to create a 500 μg/mL solution. All solutions were filtered through a PTFE membrane filter (0.45 μm). Rosmarinic acid was detected after 12 min. The rosmarinic acid content in MOLE was calculated from the peak area of the sample solution. A calibration curve (*R* ^2^ = 1.0000) was constructed using the peak areas and concentrations of three standard solutions.

### 2.8 The Addition of a Conditioned Medium (CM) From UVB‐Irradiated Keratinocytes to Fibroblasts

After overnight incubation of HaCaT keratinocytes seeded at 1.0 × 10^6^ cells/dish in a 100 mm^2^ dish in DMEM containing 10% FBS, the medium was replaced with HBSS, and the cells were irradiated with UVB (TOREX FL20S E‐30/DMR 20 W, Toshiba Medical Systems, Tochigi, Japan) at a dose of 75 mJ/cm^2^. After UVB irradiation, cells were cultured in fresh serum‐free DMEM for 24 h. The supernatant of the centrifuged culture medium from HaCaT keratinocytes was used as the CM for UVB‐exposed keratinocytes. The production of Endo180 and type I collagen from fibroblasts cultured in serum‐free DMEM containing MOLE for 24 h prior to treatment with the CM of UVB‐exposed keratinocytes for 48 h was quantified using the ELISA method described above or below.

### 2.9 Type I Collagen ELISA

The supernatant of the culture medium from NB1RGB cells was added to the ELISA plate and incubated overnight at 4°C. The wells were incubated with anti‐human rabbit type I collagen antibody for 2 h at 37°C after blocking with 1% BSA‐PBS. The wells were further incubated with HRP‐conjugated anti‐rabbit IgG antibody for 1 h at 37°C. Type I collagen was stained with ABTS as the substrate for HRP. The absorbance at 405 nm was measured using a microplate reader. A calibration curve was prepared using recombinant type I collagen protein and used to calculate type I collagen concentration per cell. The concentration of type I collagen was expressed as a percentage of the level in the control cells.

### 2.10 Human Clinical Trial

A double‐blind, randomized, placebo‐controlled study was conducted to evaluate the antiwrinkle effect of MOLE. The 8‐week study involved 20 Japanese female volunteers (mean age 44.5 ± 3.4 years). The participants had eye‐wrinkle grades based on the guidelines of the Japan Cosmetic Industry Association [11] of 1.0–3.0. The inclusion and exclusion criteria are listed in Table S2. The study was commissioned by the Institute of General Health Development Co., Ltd. and was conducted under ethical considerations in accordance with the Declaration of Helsinki (as amended by the 2008 Seoul General Assembly) and based on the study protocol IWSK‐20744 that was prepared and approved on September 6, 2016. Participants received a full explanation of the study and provided informed consent in advance. All participants were tested simultaneously to avoid seasonal variations in wrinkle development due to dryness. Each subject applied approximately 50 mg of a cream formulation containing 200 μg/mL MOLE or a cream formulation without MOLE (placebo) on each side of the eye corner (crow's foot area) twice each day for 8 weeks. The creams formulated with MOLE and the placebo were indistinguishable in terms of appearance and packaging. The wrinkle grade at the eye corner was determined by visual evaluation by a trained expert, and images were captured at the beginning of the study and at 4 and 8 weeks thereafter.

### 2.11 Statistical Analysis

All data are expressed as mean ± standard error of the mean (SEM). For in vitro tests, multiple comparisons between individual treatments and controls were performed using Dunnett's test. For in vivo test, between‐group differences were assessed using the Mann–Whitney's *U* test. Statistical significance was set at *p* < 0.05.

## 3 Results

### 3.1 Screening of Materials That Promote Endo180 Production in Fibroblasts

**FIGURE 1:**
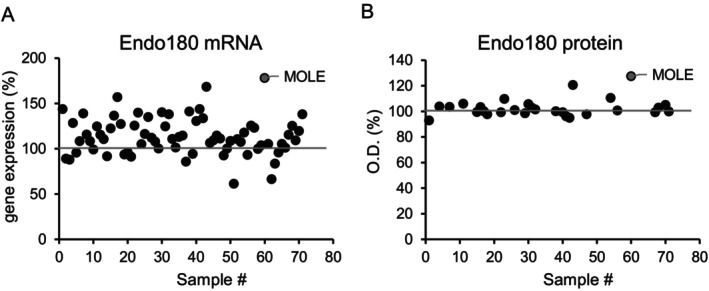
Screening of materials that promote Endo180 production in fibroblasts. (A) Seventy‐one natural extracts were screened for their promoting effect on Endo180 mRNA expression in NB1RGB cells cultured for 24 h by qRT‐PCR. (B) Twenty‐eight natural extracts were screened by ELISA for their promoting effect on Endo180 protein production in NB1RGB cells cultured for 24 h. The screening was performed in triplicate. One dot indicates one natural extract. The percentage of End180 level in nontreated cells was regarded as 100%.

A total of 71 natural extracts were screened to select materials that promoted Endo180 production in fibroblasts. As the first step, we examined mRNA expression of Endo180 in normal human dermal fibroblasts (NB1RGB cells) treated with their extract, and this resulted in the selection of 28 natural extracts that promoted the expression of mRNA by over 120% at doses of 5 or 50 μg/mL (Figure 1A). Second, we examined the protein production of Endo180 in NB1RGB cells treated with the natural extracts selected in the first step. We observed that MOLE at doses of 50 μg/mL resulted in the highest expression of both mRNA and protein at 178.1% and 127.4%, respectively (Figure 1B).

### 3.2 Promoting Effect of MOLE on Endo180 Production

**FIGURE 2:**
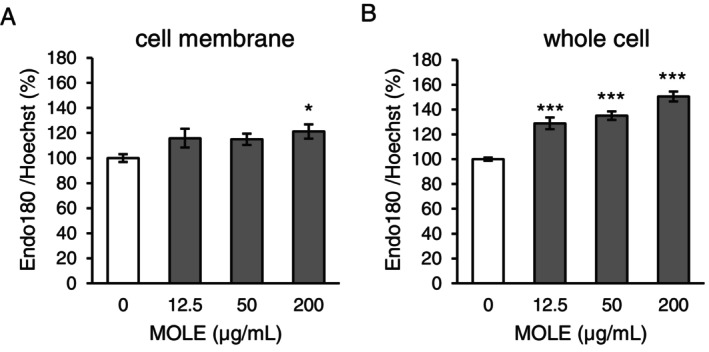
Effect of MOLE on Endo180 production in fibroblasts. NB1RGB cells treated without (control, white) or with MOLE (gray) were cultured for 24 h. Endo180 protein levels on the cell membrane (A) and in whole cells (B) were determined using ELISA. The number of cells was determined by measuring the fluorescence of the nuclei stained with Hoechst 33342. Data are expressed as mean ± SEM (*n* = 6). Statistical significance was determined using Dunnett's test. *p < 0.05 ***p < 0.001.

We confirmed the effect of MOLE on protein production of Endo180 in NB1RGB cells. MOLE significantly promoted the protein production of Endo180 in the cell membrane (Figure 2A) and whole cells (Figure 2B) in a dose‐dependent manner.

### 3.3 Rosmarinic Acid is One of the Active Compounds of MOLE

**FIGURE 3:**
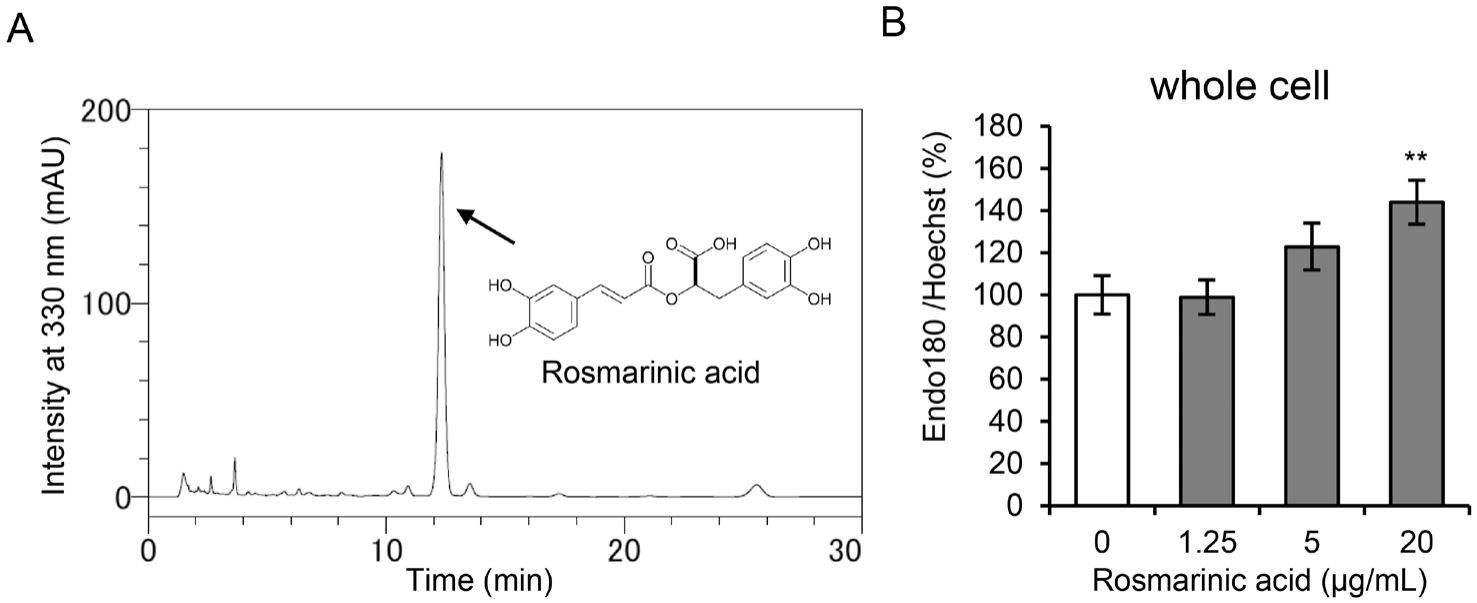
Rosmarinic acid content in MOLE and its effect on Endo180 production in fibroblasts. (A) The quantitative analysis of rosmarinic acid in MOLE was performed using HPLC, and the rosmarinic acid peak and structure are presented in the chromatogram. (B) NB1RGB cells treated without (control, white) or with rosmarinic acid (gray) were cultured for 24 h. The Endo180 protein level in the whole cell was examined by ELISA. The number of cells was determined by measuring the fluorescence of nuclei stained with Hoechst 33342. The data are expressed as mean ± SEM (*n* = 5). The statistical differences were determined using Dunnett's test. **p < 0.01.

The content of rosmarinic acid, a major component of MOLE, as analyzed by HPLC was 10.2% (Figure 3A). Rosmarinic acid significantly promoted the production of Endo180 in whole cells at doses of 20 μg/mL (Figure 3B).

### 3.4 Recovery Effect of MOLE on the Reduction of Endo180 and Type I Collagen in Fibroblasts Induced by CM of UVB‐Irradiated Keratinocytes

**FIGURE 4:**
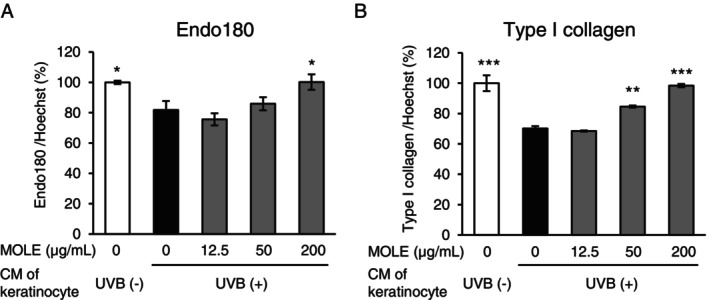
MOLE recovery effect on the reduction of Endo180 and type I collagen induced by CM of UVB‐irradiated keratinocytes in fibroblasts. HaCaT keratinocytes were irradiated with UVB at doses of 0 (UVB [−] control, white) or 75 (UVB [+] control, black) mJ/cm^2^ and cultured in fresh medium for 24 h. The supernatants were used as the CM for UVB‐exposed keratinocytes. The production of Endo180 (A) and type I collagen (B) by NB1RGB cells treated with MOLE (gray) for 24 h prior to culturing with the CM of UVB‐exposed keratinocytes for 48 h was determined using ELISA. The number of cells was determined by measuring the fluorescence of the nuclei stained with Hoechst 33342. The data are expressed as mean ± SEM (*n* = 3). Statistical significance was determined using Dunnett's test. *p < 0.05, **p < 0.01, ***p < 0.001 versus UVB (+) control.

We investigated if MOLE treatment of fibroblasts could improve the decreased Endo180 levels caused by the addition of CM of UVB‐irradiated keratinocytes and whether the increase in Endo180 could suppress the decrease in collagen production. The CM of UVB‐irradiated HaCaT keratinocytes significantly decreased Endo180 production and type I collagen production in NB1RGB cells, and MOLE treatment of NB1RGB cells significantly suppressed the decrease in Endo180 and type I collagen (Figure 4). In contrast, MOLE did not promote type I collagen production in NB1RGB cells (Figure S1).

### 3.5 Antiwrinkle Effect of MOLE at Eye Corners of Japanese Women

**FIGURE 5:**
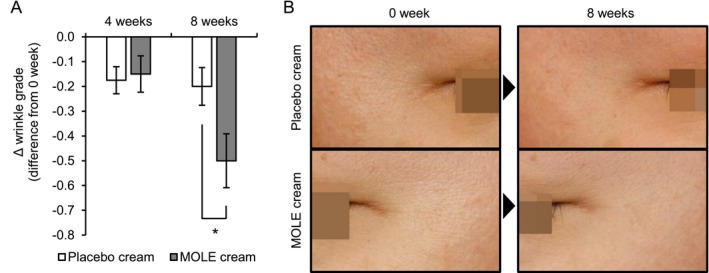
Antiwrinkle effect of the cream formulated with MOLE at the eye corner in a double‐blind, randomized, and placebo‐controlled study. The wrinkle grade at the eye corner of healthy women treated with the cream formulation containing 200 μg/mL MOLE or the placebo was determined by visual evaluation by a trained expert at 0, 4, and 8 weeks. (A) The difference in values at 4 and 8 weeks compared to those at 0 weeks. Between‐group differences were tested using the Mann–Whitney *U* test for statistical analysis. *p < 0.05. (B) Representative images of the outer eye corner were obtained at Weeks 0 and 8.

As MOLE promoted Endo180 production and suppressed the decrease in collagen in in vitro studies, we examined the antiwrinkle effect of MOLE in humans in an in vivo study. We conducted a test to measure the improvement in wrinkles at the outer eye corners of 22 healthy Japanese female volunteers with wrinkle grades of 1–3 for 8 weeks. Due to the withdrawal of two volunteers for personal reasons, the improvement effect on wrinkles was analyzed using the results of the remaining 20 volunteers. In the between‐group comparison, the difference in wrinkle grade from 0 weeks as determined by visual evaluation was significantly lower in the MOLE‐formulated cream group than it was in the placebo cream group after 8 weeks of application (Figure 5A,B).

## 4 Discussion

First, we identified natural extracts that promoted Endo180 production in terms of mRNA and protein expression in dermal fibroblasts (Figure 1). It has been reported that 20% of Endo180 is located on the plasma membrane, and the remaining 80% is located on the intracellular membrane in the cytosol, possibly as an early cell endosome [12]. If Endo180 in the cell membrane is lost by endocytosis, Endo180 is restored by protein from the vesicular membrane, thus maintaining the amount of Endo180 protein in the cell membrane [13]. Considering the role of Endo180, it is essential that Endo180 is localized in the cell membrane. Thus, it is important that materials not only promote the production of Endo180 but also translocate Endo180 to the cell membrane. Indeed, MOLE that was chosen based on the screening promoted the production of Endo180 in a dose‐dependent manner and translocated Endo180 to the cell membrane of dermal fibroblasts (Figure 2). This suggests that MOLE normalizes collagen remodeling via Endo180 supplementation in the cell membrane.

*M. officinalis* , also known as lemon balm, belongs to the Lamiaceae family and has been used as a traditional herb for indigestion, insomnia, and anxiety in Europe as well as for medicinal and cosmetic purposes [14, 15, 16]. However, the effect of lemon balm on Endo180 expression has not been reported. Lemon balm leaves have been reported to contain phenolic and flavonoid components such as caffeic acid, gallic acid, ellagic acid, chlorogenic acid, rutin, quercetin, and luteolin‐3'‐O‐glucuronide in addition to rosmarinic acid [17, 18]. We next sought to identify the active component in MOLE while focusing on rosmarinic acid that is present at a concentration of 10% in MOLE (Figure 3A). Rosmarinic acid caused a significant promotion of Endo180 production in whole cells at 20 μg/mL (corresponding to MOLE at a concentration of 200 μg/mL) and failed to cause significant promotion at concentrations of 1.25 or 5 μg/mL (Figure 3B). These results indicate that rosmarinic acid is one of the active components, but other polyphenolic components in MOLE may also contribute to this effect. To the best of our knowledge, this is the first study to report the stimulatory effect of MOLE on Endo180 production. However, we have not determined how MOLE and rosmarinic acid act or what signaling pathways are involved in Endo180 production. These issues should be addressed in future studies.

Considering the role of Endo180 in collagen metabolism in which Endo180 supplies amino acids used for the molecular synthesis of collagen through uptake of collagen fragments, it is expected that maintaining the level of Endo180 in fibroblasts will lead to the maintenance of collagen synthesis. In the previous study, we reported that interleukin 1α secreted from UVB‐irradiated keratinocytes decreases Endo180 in dermal fibroblasts [9]. Thus, it may be effective to increase the basal level of Endo180 by promoting its production to suppress the decrease in Endo180 caused by keratinocyte‐derived cytokines. MOLE maintained the level of Endo180 in fibroblasts by suppressing the decrease caused by the CM of UVB‐irradiated keratinocytes and concomitantly maintained the synthesis of type I collagen (Figure 4). On the other hand, MOLE did not promote type I collagen production in fibroblasts (Figure S1). Thus, these facts indicate that increasing Endo180 in fibroblasts is an effective pathway for improving skin aging by maintaining the amounts of type I collagen.

Based on these in vitro results, it was expected that MOLE would improve wrinkles in human skin. The application of MOLE‐containing cream for 8 weeks showed significant improvement in wrinkle grade compared to that of the placebo cream (Figure 5).

**FIGURE 6:**
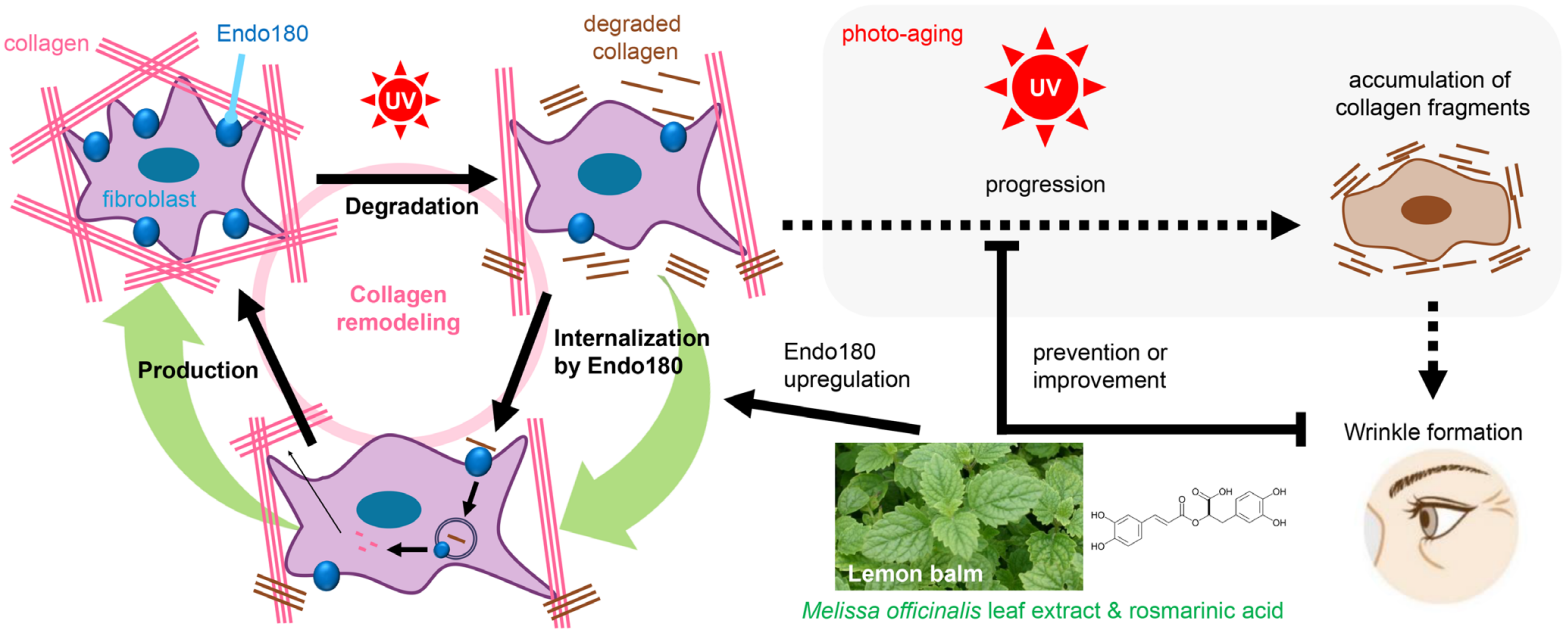
Model for antiwrinkle effect of *Melissa officinalis* leaf extract on human skin.

In conclusion, promoting the production of Endo180 in fibroblasts is one effective pathway for realizing an antiwrinkle effect through maintaining collagen levels, and it was found that MOLE possessed excellent effects in promoting Endo180 production (Figure 6).

## Author Contributions

All authors contributed to the conception and design of this study. Material preparation, data collection, and analyses were performed by H.I. and Y.K. H.I. wrote the first draft of the manuscript. H.M. and A.T. reviewed and edited the manuscript. All authors have read and approved the final version of the manuscript.

## Ethics Statement

All procedures involving human participants performed in this study were in accordance with the ethical standards of the institutional research committee and with the 1964 Helsinki Declaration and its later amendments or comparable ethical standards.

## Consent

Informed consent was obtained from all the participants. Participants signed an informed consent form regarding the publication of their data and photographs.

## Conflicts of Interest

The authors declare no conflicts of interest.

## Supporting information


Data S1.

## Data Availability

The datasets generated and/or analyzed in the current study are available from the corresponding author upon reasonable request.
