# Supplementary material for: Lemon Balm ( Melissa officinalis L.) Leaf Extract Promotes Endo180 Production in Dermal Fibroblasts and has Antiwrinkle Effect on Human Skin
Source: Photodermatol Photoimmunol Photomed. 2025 Jan 31;41(2):e70006. doi: 10.1111/phpp.70006 (PMC11785151; doi:10.1111/phpp.70006)
Supplement: Supplementary file 1 — Data S1. [file PHPP-41-e70006-s001.docx]

Supporting Information

***Melissa officinalis* L. leaf extract promotes Endo180 production in dermal fibroblasts and has anti-wrinkle effect on human skin**

Hiroyasu Iwahashi*, Yoshihito Kawashima, Hitoshi Masaki, Atsushi Taga

*Correspondence: E-mail address: [h-iwahashi@maruzenpcy.co.jp](mailto:h-iwahashi@maruzenpcy.co.jp)

**Contents**

**FIGURE S1** Direct effect of MOLE on type I collagen production in fibroblasts

**TABLE S1** List of natural extracts for screening

**TABLE S2** Inclusion and exclusion criteria in human clinical trial

**
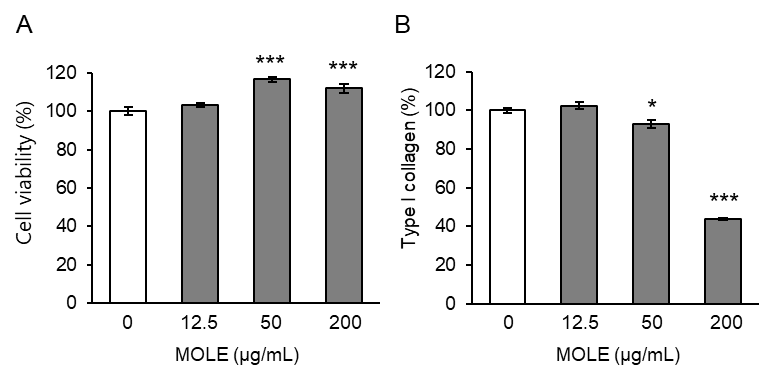
**

**FIGURE S1** Direct effect of MOLE on type I collagen production in fibroblasts

NB1RGB cells treated without (control, white) or with the indicated concentration of MOLE (gray) were cultured for 3 days. (A) Cell viability was determined using MTT assay. (B) The production of type I collagen was determined by ELISA. The result was expressed as a percentage of that in control cells. The data are expressed as mean ± SEM (n = 5). Statistical differences were determined using Dunnett’s test. *:*p* < 0.05, **:*p* < 0.01, ***:*p* < 0.001. MOLE, *Melissa officinalis* leaf extract.

**TABLE S1** List of natural extracts for screening

| No. | Scientific name | Part of natural product |
| --- | --- | --- |
| 1 | *Uncaria gambir* (W.Hunter) Roxb. | Leaf/scion |
| 2 | *Hydrangea serrata* (Thunb.) Ser. var. *thunbergii* (Siebold.) H.Ohba | Leaf |
| 3 | *Arnica montana* L. | Flower |
| 4 | *Aloe ferox* Mill. | Leaf |
| 5 | *Ginkgo biloba* L. | Leaf |
| 6 | *Urtica dioica* L. | Leaf |
| 7 | *Foeniculum vulgare* Miller | Fruit |
| 8 | *Malva sylvestris* L. | Flower |
| 9 | *Isodonis japonicus* (Burm.f.) H.Hara | Leaf/Stalk |
| 10 | *Hypericum perforatum* L. | Flower/Leaf/Stem |
| 11 | *Artemisia indica* Willd. var. *maximowiczii* (Nakai) H.Hara | Leaf |
| 12 | *Matricaria chamomila* L. | Flower |
| 13 | *Glycyrrhiza glabra* L. | Root |
| 14 | *Rubus idaeus* L. | Fruit |
| 15 | *Prunus armeniaca* L. | Kernel |
| 16 | *Lonicera japonica* Thunb. | Flower |
| 17 | *Osmanthus fragrans* Lour. | Flower |
| 18 | *Sophora flavescens* Aiton | Root |
| 19 | *Sasa veitchii* (Carrière) Rehder | Leaf |
| 20 | *Citrus paradisi* Macfad. | Fruit |
| 21 | *Cinnamomum cassia* (L.) J.Presl | Bark |
| 22 | *Alpinia speciosa* (Wendl.) K. Schum. | Leaf |
| 23 | *Gentiana lutea* L. | Rhizome/Root |
| 24 | *Geranium thunbergii* Siebold ex Lindl. & Paxton | Flower/Leaf/Stem |
| 25 | *Saccharomyces cerevisiae* Meyer ex E. C. Hansen | Bacterial cell |
| 26 | *Arctium lappa* L. | Root |
| 27 | *Oryza sativa* L. | Bran |
| 28 | *Salvia officinalis* L. | Leaf |
| 29 | *Gardenia jasminoides* J.Ellis | Fruit |
| 30 | *Zanthoxylum piperitum* (L.) DC. | Peel |
| 31 | *Lentinula edodes* (Berk.) Pegler | Fruiting body |
| 32 | *Rehmannia glutinosa* Libosch. var. *purpurea* Makino | Root |
| 33 | *Perilla frutescens* (L.) Britton var. *crispa* (Benth.) W.Deane | Leaf |
| 34 | *Houttuynia cordata* Thunb. | Above-ground part |
| 35 | *Sanguisorba officinalis* L. | Root |
| 36 | *Acorus calamus* L. var. *angustatus* Besser | Rhizome |
| 37 | *Betula pendula* Roth | Bark |
| 38 | *Equisetum arvense* L. | Whole plant |
| 39 | *Averrhoa carambola* L. | Leaf |
| 40 | *Achillea millefolium* L. | Whole plant |
| 41 | *Cnidium officinale* Makino | Rhizome |
| 42 | *Morus alba* L. | Root |
| 43 | *Ziziphus jujuba* Mill. var. *inermis* (Bunge) Rehder | Fruit |
| 44 | *Syzygium aromaticum* (L.) Merr. & L.M.Perry (synonym:*Eugenia caryophyllata* Thunb.) | Flower |
| 45 | *Citrus unshiu* (Swingle) Marcow. | Peel |
| 46 | *Calendula officinalis* L. | Flower |
| 47 | *Angelica acutiloba* (Siebold & Zucc.) Kitag. | Root |
| 48 | *Prunus persica* (L.) Batsch | Kernel |
| 49 | *Panax ginseng* C.A.Mey. | Root |
| 50 | *Lonicera japonica* Thunb. | Leaf |
| 51 | *Rubus ellipticus* Smith | Root |
| 52 | *Wolfiporia cocos* (Schwein.) Ryv. & Gilbn. (synonym:*Poria cocos* (Schwein.) F.A.Wolf) | Sclerotium |
| 53 | *Ruscus aculeatus* L. | Root |
| 54 | *Prunus domestica* L. | Fruit |
| 55 | *Luffa cylindrica* (L.) M.Roem. | Fruit/Leaf/Stem |
| 56 | *Carthamus tinctorius* L. | Flower |
| 57 | *Mentha × piperita* L. | Leaf |
| 58 | *Paeonia suffruticosa* Andrews | Root |
| 59 | *Humulus lupulus* L. | Spikes |
| 60 | *Melissa officinalis* L. | Leaf |
| 61 | *Prunus persica* (L.) Batsch | Leaf |
| 62 | *Eucalyptus globulus* Labill. | Leaf |
| 63 | *Saxifraga stolonifera* Curtis | Whole plant |
| 64 | *Citrus junos* Siebold ex Tanaka | Fruit |
| 65 | *Lilium candidum* L. | Bulb |
| 66 | *Coix lacryma-jobi* L. var. *ma-yuen* (Rom.Caill.) Stapf | Seed |
| 67 | *Lavandula angustifolia* Mill. | Flower |
| 68 | *Camellia sinensis* (L.) Kuntze | Leaf |
| 69 | *Rosmarinus officinalis* L. | Leaf |
| 70 | *Apis mellifera* Linnaeus | Royal jelly |
| 71 | *Thymus serpy+C2:F73llum* L. | Above-ground part |

**TABLE S2** Inclusion and exclusion criteria in human clinical trial

| The inclusion criteria | The exclusion criteria |
| --- | --- |
| 1. Japanese women who are 35 to 50 years old at the time of consent 2. participants must have wrinkles mainly at the corners of the left and right eyes with a wrinkle grade of 1 to 3 3. the wrinkle grade of the left and right eye corner is the same (a difference of up to 1.0 is acceptable) 4. the left and right eye lids must be free of scars, warts, pimples, burns, etc., that would affect the evaluation measurement. | 1. participants with a history of allergy to cosmetics 2. participants undergoing "hormone replacement therapy" 3. participants with a history of aesthetic medicine that affects the study site 4. other participants deemed inappropriate by the physician involved in the study 5. participants who are currently undergoing dermatological treatment 6. pregnant, possibly pregnant, or lactating 7. participants who are participating in other clinical trials. |
